# ACTH Antagonists

**DOI:** 10.3389/fendo.2016.00101

**Published:** 2016-08-05

**Authors:** Adrian John Clark, Rachel Forfar, Mashal Hussain, Jeff Jerman, Ed McIver, Debra Taylor, Li Chan

**Affiliations:** ^1^Centre for Endocrinology, William Harvey Research Institute, Queen Mary University of London, London, UK; ^2^Centre for Therapeutics Discovery, MRC Technology, Stevenage, UK

**Keywords:** adrenocorticotropin hormone, receptor antagonism, Cushing’s syndrome, congenital adrenal hyperplasia, high throughput screening, G protein-coupled receptor, receptor modelling, peptide hormone antagonists

## Abstract

Adrenocorticotropin (ACTH) acts via a highly selective receptor that is a member of the melanocortin receptor subfamily of type 1 G protein-coupled receptors. The ACTH receptor, also known as the melanocortin 2 receptor (MC2R), is unusual in that it is absolutely dependent on a small accessory protein, melanocortin receptor accessory protein (MRAP) for cell surface expression and function. ACTH is the only known naturally occurring agonist for this receptor. This lack of redundancy and high degree of ligand specificity suggests that antagonism of this receptor could provide a useful therapeutic aid and a potential investigational tool. Clinical situations in which this could be useful include (1) Cushing’s disease and ectopic ACTH syndrome – especially while preparing for definitive treatment of a causative tumor, or in refractory cases, or (2) congenital adrenal hyperplasia – as an adjunct to glucocorticoid replacement. A case for antagonism in other clinical situations in which there is ACTH excess can also be made. In this article, we will explore the scientific and clinical case for an ACTH antagonist, and will review the evidence for existing and recently described peptides and modified peptides in this role.

## Introduction

The impact of receptor antagonism on modern medicine cannot be understated. Classical examples include the β-blockers in the treatment of hypertension and cardiovascular disease (1) and histamine H2 antagonism in the treatment of gastric hyperacidity (2). Even in the field of endocrinology, receptor antagonism of steroid hormones [e.g., tamoxifen (3), eplerenone (4), and flutamide (5)] and some peptide hormones [e.g., pegvisomant (6) and conivaptan (7)] has had major life-changing impact. The pituitary–adrenal axis is one endocrine axis that when disrupted can be associated with a wide range of pathologies, and yet, despite the fact that it comprises several unique and thus highly targetable components, receptor antagonism has received little attention as a therapeutic approach.

In this article, we will examine the possible benefits of development of an effective antagonist to a key component of this axis, the peptide hormone adrenocorticotropin (ACTH). The disorders in which clinical benefit might be attained will be considered. We will then consider the nature of the target – ACTH and the ACTH receptor complex, and certain unique features before discussing the history of ACTH antagonist research, ending with a description of the current state-of-the art. Initially, a brief description of the pituitary–adrenal axis and its key components is necessary.

## The Pituitary–Adrenal Axis

The corticotroph cells of the anterior pituitary gland are responsible for synthesis and secretion of the 39 residue peptide, ACTH (8). ACTH is derived from a larger precursor protein, pro-opiomelanocortin (POMC), by the action of a specific pro-hormone convertase enzyme (PC1 or PCSK1) (9). In other tissues – for example, the hypothalamus – this precursor is processed differently to produce α-MSH instead of ACTH (10). ACTH is synthesized and secreted by the pituitary in response to tonic control from the hypothalamus – principally in the form of two peptide hormones – corticotrophin-releasing hormone (CRH) and vasopressin (AVP), which in turn are regulated by multiple higher factors including stress (11).

Adrenocorticotropin has a short half-life in the circulation (12) and acts on a highly specific G protein-coupled receptor expressed almost uniquely in the adrenal cortex (13). This receptor, the MC2R is one of five members of the melanocortin receptor family – see Table 1. ACTH can activate all five of these receptors, although at physiological circulating levels, the sensitivity of the other receptors is such that they are not activated. Importantly, the naturally occurring agonists for these other receptors – α-MSH, γ-MSH, and possibly β-MSH – have no affinity for the MC2R (14, 15). Thus the MC2R is a highly sensitive and highly specific receptor for ACTH with a major, essential function of stimulating the fasciculata cells of the adrenal cortex to synthesize and secrete glucocorticoid. In addition, ACTH can stimulate zona glomerulosa cells to secrete mineralocorticoid and zona reticularis cells to secrete adrenal androgens.

**Table 1:** **A summary of the main features of each of the melanocortin receptors in the human**.

	Major sites of expression	Ligand preference	Function	Effect of deletion	Comments
MC1R	Melanocytes	α-MSH > ACTH > γ-MSH	Pigmentation of hair and skin	Red hair, pale skin	Agouti antagonizes
MC2R	Adrenal cortex	ACTH	Steroidogenesis adrenal growth	Adrenal failure	Absolute dependency on MRAP
MC3R	Brain, spinal cord	γ-MSH > α-MSH = ACTH	Complex, inhibits POMC neurones	Obesity	
MC4R	Brain, spinal cord	α-MSH > ACTH > γ-MSH	Appetite regulation	Obesity	Enhanced action with MRAP2 AGRP is natural antagonist
MC5R	Multiple tissues	α-MSH > ACTH > γ-MSH	Exocrine gland function	Defective water repulsion	

Glucocorticoid (cortisol in man and most other species, corticosterone in rodents), secreted by the adrenal gland exert a plethora of physiological actions on virtually every cell in the organism. These actions are the result of interaction with the widely expressed glucocorticoid receptor – a nuclear hormone receptor. Glucocorticoid may also activate a second related receptor – the mineralocorticoid receptor – which is less widely expressed. However, the action of the 11 β-hydroxysteroid dehydrogenase type 2 enzyme inactivates glucocorticoid in mineralocorticoid receptor expressing tissues under normal circumstances leaving these receptors responsive to aldosterone (16). From an endocrine perspective, a key role of glucocorticoid is to feedback negatively on the pituitary and hypothalamus to inhibit ACTH secretion (17).

From this brief description, it can be seen that in theory, the MC2R should provide a perfect substrate for receptor targeting. This is a receptor with, effectively, a single function, expressed in a highly tissue-restricted way and activated by a single, highly specific agonist. The question is – if it were possible to design the perfect antagonist – what clinical role might it play?

## Disorders of the Pituitary–Adrenal Axis

Disorders of this axis are, fortunately, uncommon and can be subdivided into disorders of hormone deficiency and excess. Glucocorticoid deficiency seems unlikely to benefit from MC2R antagonism, but in certain specific circumstances, there could be a valuable role for this therapeutic option as discussed later.

### Glucocorticoid Excess

Glucocorticoid excess may result from primary adrenal disease – typically an adrenal adenoma or carcinoma – and is independent of ACTH. Indeed ACTH is normally suppressed by the actions of the negative feedback loop. More often, cortisol excess or Cushing’s syndrome is the result of a pituitary adenoma secreting excess ACTH – known as Cushing’s Disease – or less commonly a non-pituitary tumor that “ectopically” secretes ACTH. This group of disorders might theoretically provide a suitable target for an MC2R antagonist.

#### Cushing’s Disease

Corticotroph adenomas are small, usually slow growing, benign tumors that normally come to clinical attention as a result of the effects of glucocorticoid excess, rather than because of the physical effects of an expanding tumor. Typically, Cushing’s syndrome may take many years to develop. Consequently the diagnosis of the disorder and exclusion of other causes of Cushing’s syndrome is a significant challenge. Once a diagnosis is conclusively made, the optimal treatment is surgical removal of the tumor – ideally preserving the remaining pituitary function. Surgery for Cushing’s disease requires extensive experience and skill and is normally undertaken in specialist centers (18).

**Figure 1:**
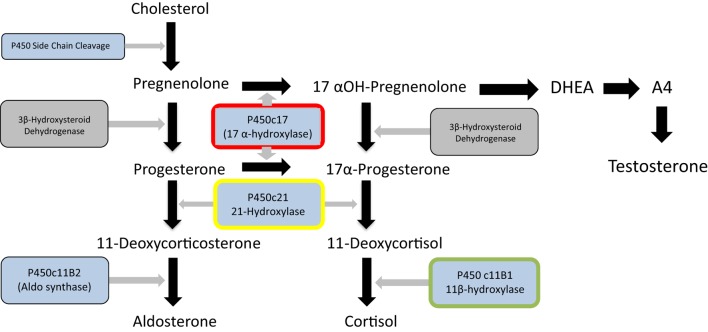
**Major steroid synthetic pathways in the human showing the three main end products – cortisol, testosterone and aldosterone, the key intermediates, and the main enzymes**. 21-Hydroxylase deficiency (enzyme highlighted in yellow) is the major cause of congenital adrenal hyperplasia. It can be seen that deficiency or inhibition will result in cortisol and aldosterone deficiency and androgen excess. Inhibition of 17 α-hydroxylase (highlighted in red) by abiraterone in contrast will lead to cortisol and testosterone deficiency and overproduction of aldosterone. Metyrapone inhibits 11 β-hydroxylase (highlighted in green) and this may lead to an overproduction of adrenal androgens. The p450 inhibitor, ketoconazole will impair the action of all these enzymes and other P450 enzymes (shown in blue) and thus will not result in overproduction of steroid.

In some patients, the metabolic consequences of their untreated glucocorticoid excess are so significant that there would be risks in immediately proceeding to complex or prolonged surgery. The glucocorticoid synthesis blockers metyrapone and/or ketoconazole are frequently used in this situation to reduce steroid production (see Figure 1), and most patients tolerate and respond to this treatment reasonably well (19–21). However, an MC2R antagonist could be equally effective in this situation.

Following surgery, the glucocorticoid excess will come under rapid control in a minority of patients. More frequently, there will be a reduction in steroid over-secretion that may tail off over several weeks. In other cases, it may be necessary to re-explore the pituitary surgically, and this may result in pituitary clearance with a loss of other pituitary hormones (18). Control of glucocorticoid excess during this interim period will often necessitate the use of metyrapone and/or ketoconazole.

If further surgical measures are unsuccessful, endocrinologists may turn to the somatostatin receptor 5 agonist, pasireotide, which directly targets the corticotroph adenoma and provides partial or complete control of Cushing’s in a proportion of cases (22). Other second-line options in this situation include pituitary radiotherapy or adrenalectomy. The former may take several years to normalize the glucocorticoid excess, necessitating metyrapone and/or ketoconazole during this time. An MC2R antagonist could be a possible alternative.

While glucocorticoid synthesis inhibitors are usually effective and reasonably well tolerated in these situations, there are some potential disadvantages to their prolonged use. Metyrapone blocks the 11β-hydroxylase enzyme, required for the last step of cortisol synthesis from 11-deoxycortisol. Consequently, as shown in Figure 1, steroid precursors are channeled through the androgen pathways resulting in increased secretion of adrenal androgens. In women, this may induce hirsutism and in pre-pubertal children both virilization and early puberty (23, 24). Ketoconazole, a cytochrome P450 inhibitor, inhibits several steps of steroidogenesis and does not usually cause androgen excess. However, complications include potential rare but serious hepatotoxicity and multiple drug interactions. Consequently, although these treatments are effective and inexpensive, there could be a place for a highly specific MC2R antagonist in the management of Cushing’s disease.

#### Ectopic ACTH Syndrome

The mechanisms of ectopic ACTH syndrome are essentially the same as those of Cushing’s disease except that the underlying tumor is outside the pituitary gland. These rare tumors are often small carcinoid tumors that may occur anywhere in the lungs and gastrointestinal tract. Improvements in imaging over the last 10–20 years together with intravenous sampling for ACTH have made identification of the primary source far simpler. Once identified, and if surgical removal of the underlying tumor is possible, then this can be curative. As with Cushing’s disease, it may be helpful to use a cortisol synthesis blocking drug while waiting for definitive treatment (18) and in cases where definitive treatment is not possible. Hence, for the same reasons described above, an MC2R antagonist may have a place in the clinical management of such conditions.

Small cell lung cancer makes up about 20% of all lung cancer is a highly malignant neuroendocrine tumor of poor prognosis. This tumor is frequently associated with ectopic ACTH secretion, and the development of Cushing’s syndrome in this disease may obviously worsen prognosis. Thus there may also be value in using an ACTH antagonist in this clinical situation.

### Glucocorticoid Deficiency

The second group of disorders in which there may be an place for antagonizing ACTH action are those in which the physiological negative feedback of cortisol is reduced or lost, leading to a compensatory increase in ACTH secretion. In this situation, it is the stimulation of non-glucocorticoid adrenal steroidogenesis by ACTH, which requires control.

#### Congenital Adrenal Hyperplasia

The most prominent example of such a situation is that of congenital adrenal hyperplasia, caused in the majority of cases by mutations in both alleles of the *CYP21* gene encoding the 21-hydroxylase enzyme necessary for the penultimate step of cortisol synthesis (Figure 1) (25, 26). This is one of the commonest human autosomal recessive disorders occurring in about 1 in 15,000 live births. Reduced glucocorticoid feedback results in ACTH stimulation of adrenal androgen production. As a consequence, affected female children are likely to be virilized or have ambiguous genitalia at presentation. Life-long treatment with glucocorticoids will restore any cortisol deficiency and suppress ACTH secretion and subsequent androgen production (27). However, achieving the optimal dose and timings of hydrocortisone replacement to avoid the adverse effects of excessive glucocorticoid on growth and metabolism while maintaining adequate androgen suppression is challenging, resulting in poor health outcomes (28). Availability of an easily administered ACTH antagonist would be likely to facilitate treatment, allowing a “block and replace” approach in which the physician could focus on treatment of glucocorticoid replacement alone rather than androgen suppression.

#### Prostate Cancer Treatment

The use of the drug Abiratarone in the treatment of prostate cancer induces a form of acquired adrenal hyperplasia. Abiratarone is a potent inhibitor of the 17 α-hydroxylase and 17,20 lyase enzymes in the adrenal and is used to very effectively reduce the production of adrenal androgens in castration-resistant prostate cancer, with valuable benefits to prostate cancer treatment (29). Examination of adrenal steroid synthetic pathways (Figure 1) demonstrates that this inhibition is likely to channel steroid synthesis toward deoxycorticosterone synthesis and aldosterone production, leading to mineralocorticoid excess and glucocorticoid deficiency. The latter requires glucocorticoid replacement, but the fluid overload, hypertension, and hypokalemia resulting from aldosterone excess require treatment with a mineralocorticoid antagonist such as eplerenone (30). Use of an ACTH antagonist together with a replacement dose of hydrocortisone may be a preferred approach in this situation.

### Investigation of Endocrine Disease

A further potential use of an ACTH antagonist is in the investigation of adrenal disorders. One of the key questions in the investigation of Cushing’s syndrome is whether the cortisol excess is ACTH dependent. A number of tests have been used to determine this including the dexamethasone suppression test and the CRH stimulation test, combined with measurements of plasma ACTH and imaging studies. Hypoglycemic stress tests and metyrapone tests may also be required in complex cases (31). It is conceivable that the use of a single dose ACTH antagonist test could provide a simple and clear solution to this question, although it is more likely that its use in combination with other investigations would be required in most cases.

## The Target

As discussed in the Section “Introduction,” the receptor for ACTH presents a remarkably attractive target for pharmacological manipulation. It is highly specific for a single peptide agonist – ACTH [1–39], and has no affinity or response to any other naturally occurring agonist. It is expressed in functional quantities only in the adrenal cortex, and thus the possibility of unwanted off-target effects of an antagonist is unlikely.

The key component of the ACTH receptor complex is the seven transmembrane domain MC2R – perhaps surprisingly, the smallest of all the G protein-coupled receptor (GPCR) family at only 289 residues in length (32). The MC2R cannot function alone as an ACTH receptor, which led to many difficulties in its characterization after initial cloning (33). The discovery that deficiency of a small, single transmembrane domain protein caused a clinical syndrome essentially identical to that caused by MC2R deficiency led to the identification of the melanocortin 2 receptor accessory protein (MRAP) as the MC2R co-receptor (34, 35).

**Figure 2:**
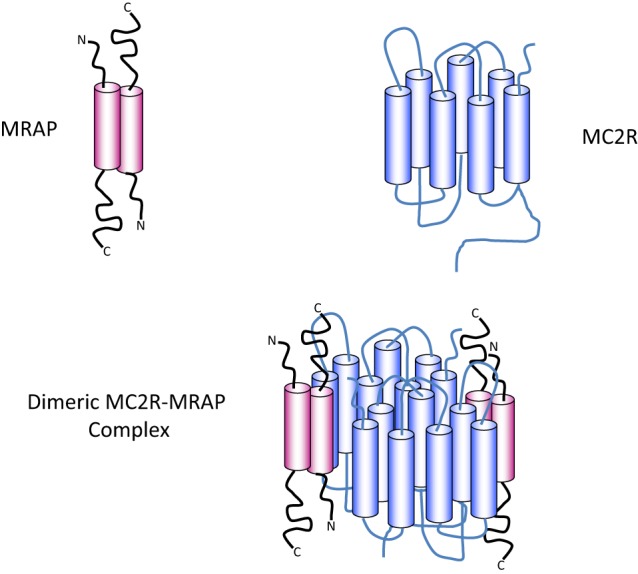
**Diagrammatic representation of the components of the ACTH receptor complex**. MRAP (pink) exists as an antiparallel homodimer with one short N-terminus and one longer C-terminus on either side of the plasma membrane. The cylindrical components represent the α-helical transmembrane domains. MC2R (blue) contains seven transmembrane domains (blue cylinders) with an N-terminal extracellular domain and a C-terminal intracellular domain. Evidence suggests that one MRAP homodimer associates with one MC2R molecule and that MC2R probably exist as homodimers as shown in the lower panel, or possibly as higher order multimers.

Melanocortin receptor accessory protein is a highly unusual protein in that it naturally exists as an antiparallel homodimer and seems to be necessary for trafficking and cell surface expression of the MC2R, as well as binding of ACTH and hence signal transduction (36, 37). In common, with many other GPCRs, MC2R has the potential to homodimerise and the evidence suggests that it exists as a homodimer with two MRAP molecules, in an antiparallel homodimer formation, associated with each MC2R component (38) (Figure 2).

**Figure 3:**
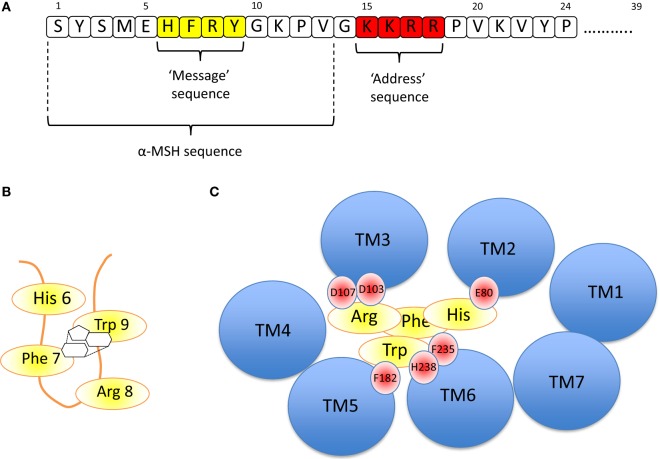
**(A)** Amino acid sequence of ACTH [1-24] using the single letter amino acid code. Note – the naturally occurring peptide is 39 residues in length. The key functional domains are the “message” sequence (yellow), which is required for activation of all the melanocortin receptors, and the “address” sequence (red), which enables only ACTH to activate the MC2R. α-MSH is equivalent to the first 13 residues of ACTH. **(B)** The “message” sequence folds into a β-hairpin loop in which the aromatic amino acids Phe 7 and Trp 9 interact with each other *via* their phenyl and indole rings, respectively, as shown. **(C)** If the MC2R is viewed from above, each of the transmembrane domains is seen as a blue circle (labeled TM1, TM2, etc). By extrapolation from modeling data from the MC4R (41) and from the MC5R (42), it seems likely that His 6 of ACTH interacts with E80 in TM2 of the MC2R, and Arg 8 interacts with D103 and 107 (of MC2R). Phe 7 and Trp 9 interact with multiple residues including F182, F235, and H238 of MC2R.

The nature of the ligand, ACTH is important in understanding receptor function (Figure 3A). The strongly conserved N-terminal 24 residues of ACTH are almost as efficient as the 39 residue naturally occurring peptide in activating this receptor. Further truncation of ACTH from the C-terminus is associated with gradual loss of activity until removal of the four basic residues (Lys–Lys–Arg–Arg) in positions 15–18, which inactivates this peptide at the ACTH receptor (39, 40). The first 13 residues are however active at all the other melanocortin receptors and thus it seems that this “tetrabasic” region acts as a “key” to unlock the MC2R–MRAP complex.

The evidence suggests that once the receptor is “unlocked,” the N-terminal region is an effective agonist for the receptor. As with all the melanocortin receptors, the His–Phe–Arg–Trp sequence (or HFRW sequence using the single letter amino acid code) at positions 6–9 and to some extent those residues flanking this induce the conformational changes required to activate the receptor. This HFRW sequence is fundamental to activation of all the melanocortin receptors and can be considered the “message” region of the peptide (40, 43, 44). Interestingly, a naturally occurring human mutation of Arg 8 in the HFRW sequence results in biologically inactive ACTH (45). The most N-terminal region (Ser–Tyr–Ser) has been reported to potentiate the action of the HFRW sequence (46).

Great strides have been made in recent years in understanding the three dimensional nature of GPCRs, based around a growing number of receptor crystal structures. No melanocortin receptor crystal structure has yet been reported, but increasingly sophisticated modeling exercises combined with receptor mutagenesis and substitution studies are providing information on how ligands interact with their receptor.

Pogozheva et al. studied the MC4R-binding site for NDP-MSH (a highly potent analog of α-MSH) and two small molecule agonists using a combination of alanine scanning mutagenesis of the receptor followed by functional analysis and *in silico* modeling. They concluded that the HFRW sequence of NDP-MSH was required to form a β-hairpin-like structure so that the phenyl ring of Phe 7 interacts with the indole ring of Trp 9 (Figure 3B). This allows interaction between His 6 of NDP-MSH and Glu 100 in transmembrane domain 2 (TM2) of MC4R, and Arg 8 of NDP-MSH and Asp 115 and 119 inTM3 of MC4R. The interacting aromatic residues of this sequence, Phe 7 and Trp 9 interact with the aromatic Phe at positions 261 and His 264 of MC4R in TM6 (41).

A relatively similar picture of NDP-MSH binding to the MC5R was constructed by Yang and colleagues using site-directed mutagenesis and structural modeling. They also suggested that Asp 115 and 119 in TMD 3 interact with Arg 8 of the HFRW sequence and that Phe 195 (in TMD 5) and Phe 254 (TMD 6) interact with Phe 7 and Trp 9 of HFRW. All of these residues are conserved in the MC2R (and other melanocortin receptors), and it seems highly likely that these interactions are critical in determining the HFRW binding and activation of this receptor (42). Indeed, naturally occurring homozygous mutations of Asp 103 and 107 in MC2R, the equivalent conserved Asp residues in this receptor, lead to ACTH resistance/Familial Glucocorticoid Deficiency (47). This arrangement is shown in Figure 3C.

Using a receptor chimera approach in which regions of the MC4R were substituted into the MC2R, Fridmanis et al. suggested that one of the MRAP molecules binds to MC2R in the region of transmembrane domains 4 and 5 to create a binding pocket for the tetrabasic “address” sequence in ACTH. Following this interaction, a conformational shift in the receptor transmembrane domains takes place, which permits the formation of the HFRW-binding pocket (14). Although this remains speculative, it is an attractive hypothesis. It is notable that Malik et al. have shown that it is the N-terminal region of the MRAP molecule that is required on the extracellular surface of the cell for ACTH binding (48). Clearly complete understanding of this complex area will ultimately require determination of a crystal structure of the MC2R–MRAP–ACTH complex.

## Approaches to Antagonizing ACTH

Given the extensive knowledge of the interaction of ACTH with its receptor gained over about 50 years one might anticipate that it would be a relatively straightforward matter to design an ACTH-like peptide with antagonist properties. The first attempts to do this resulted in peptides that retained the tetrabasic address region, but lacked the HFRW message sequence. This led to the development of ACTH [11–24] (49, 50) as a potential receptor antagonist. Li et al. isolated a naturally occurring peptide, ACTH [8–39], from human pituitary, which they showed to have ACTH antagonistic effects *in vitro*, and they called this corticotrophin-inhibiting peptide (CIP) (51). However, the data with each of these potential antagonists has been confusing with discrepant results for steroidogenesis and cAMP generation in some cases. For example, Szalay demonstrated that ACTH [11–24] stimulated steroidogenesis in dispersed zona glomerulosa and zona fasciculata cells (52), and Goverde and Smals (53) demonstrated some steroidogenesis with this peptide.

More recently, Kovalitskaia et al. investigated the binding of a wide range of ACTH fragments derived from an ACTH [11–24] parent peptide. They reported that the ACTH [15–18] tetrabasic fragment alone was an effective competitor for ACTH [11–24] in ligand-binding assays, and that it also failed to stimulate cAMP generation in adrenocortical membranes (54). Its use in competition with ACTH in cAMP generation or steroidogenesis has not been reported.

The consensus from most researchers seems to be that ACTH [11–24] is not an effective ACTH antagonist. This may be because the interaction between the tetrabasic region of ACTH converts the MC2R into a “primed receptor with an unoccupied HFRW-binding site, which may then be activated by the natural agonist.” Hoffman therefore used a different approach and developed an analog in which the Trp residue at position 9 of the HFRW message sequence was substituted with Phe or *N*-methyl Trp, and showed inhibition of ACTH stimulated cAMP generation on bovine adrenal membranes (55).

Liang et al. has described a number of peptide analogs of ACTH based on alanine and histidine substitutions around the HFRW region and in the spacing between this and the tetrabasic region (56). In this work, they described the marked reduction in MC2R activation observed with some of these peptides and, in a US patent filed the preceeding year, they reported potent ACTH antagonism with an ACTH [15–24] decapeptide (57).

In all of the above studies, the actions on adrenal tissues, slices, cells, or membranes have been studied, but little or no data on the selectivity of these peptide antagonists for the MC2R, or even melanocortin receptors in general have been obtained. It is usually highly desirable that any receptor active drug used therapeutically is selective for its target receptor and lacks off-target effects. The functions of the other melanocortin receptors and the effects of antagonizing or deleting them are summarized in Table 1, and it can be seen that a non-selective agent could induce a number of unwanted effects.

Bouw and colleagues reported an approach in which ACTH peptides that retain the intact tetrabasic region were substituted at various positions in the HFRW sequence and in some cases were cyclized in order to enhance stability. HEK293 cells stably expressing human MC2R and MRAP were used, and cAMP production was measured with a luminescence assay. Several peptides exhibited significant antagonist actions among which GPS1573 – a variant of ACTH [7–18] with an N-terminal nor leucine – proline sequence and d-Phe and dd-Trp (in place of the l-Phe and l-Trp) in the HFRW sequence, and a cyclized variant of this – GPS1574 were most potent (IC50s of 66 ± 23 nM and 260 ± 1 nM, respectively). These peptides retain some antagonist effect on the MC3R, MC4R and MC5R at approximately an order of magnitude less than that on the MC2R (58).

In work published in this issue, Nensey et al. report the actions of these same analogs on rat adrenal cells and show inhibitory effects in which the dose responses to ACTH [1–39] were shifted to the right by one log order or more. They also conducted *in vivo* experiments in young rats but were unable to show inhibition of the ACTH response even at 400-fold molar excess of antagonist in the case of GPS1573. GPS1574 was partially inhibitory at 30 min after ACTH injection (59).

In a recent study, presented in abstract form, researchers from Ipsen Bioscience Inc. reported the development of an ACTH-related peptide, IRC-274. This peptide was shown to inhibit ACTH binding to the human MC2R and MRAP expressed in HEK 293 cells with an IC50 of 3 nM (60). cAMP generation in response to ACTH in this same model is inhibited with an IC50 of 38 nM. Using an *in vivo* hypophysectomized rat model in which ACTH is infused by osmotic minipump, significant inhibition of corticosterone production was observed. Using a second model in which mouse AtT20 pituitary corticotroph tumor cells were implanted into athymic nude mice, inhibition of corticosterone was again observed until the implanted tumors outgrew the inhibitory action of IRC-274. Interestingly, this antagonist exhibits a high degree of selectivity for the MC2R and has no significant actions on other melanocortin receptors. The sequence and structure of this peptide have not been revealed.

## Alternative Approaches

### Small Molecules

From the above, it seems that after a rather long and chequered history some progress is now being made in developing a peptide ACTH antagonist that might ultimately be developed for use *in vivo*. The problems associated with peptide-based medication are well-known and include a short half-life in the circulation, the need to administer them by injection and the risk of inducing immunogenicity. In certain circumstances, the benefits of a peptide outweigh these potential disadvantages, and advances are being made in deriving preparations of peptides that may be taken orally or intranasally. However in many cases, there will be a need for a reliable long-term therapy as discussed earlier. Under these circumstances, it would be desirable to have an orally active agent, which would most likely be a small (non-peptide) molecule. Substantial efforts have been made to develop small molecules as agonists of the MC4 receptor with some limited success (61). With this objective in mind, we have undertaken a high throughput screen of about 200,000 small molecules using a cell-line expressing the human MC2R and MRAP, and this may provide a promising approach if a molecule with sufficient potency and selectivity can be identified.

### Antibody-Based Approaches

Humanized monoclonal antibodies directed against key signaling molecules have proven to provide effective therapeutic solutions in inflammatory diseases and cancer. This approach has been used to target ACTH and the pituitary–adrenal axis by a number of investigators and one pharmaceutical company aims to begin human studies in the near future (62). While such antibodies may not necessarily provide a long-term therapeutic solution, they seem likely to have potential in shorter-term therapeutic situations, such as around the time of pituitary surgery for Cushing’s disease.

### Corticostatins

Solomon and colleagues identified a novel lung and neutrophil peptide belonging to the defensin class of highly cationic antimicrobial peptides. They showed this peptide exhibited a number of functions including inhibition of ACTH binding and corticosterone secretion and named this corticostatin (63). It is also known as defensin α-4. These functions appear to be relatively non-specific, and little work has been published on this in recent years. We are not aware that this action of corticostatin has been explored for therapeutic purposes.

## Summary

We have reviewed the case for the development of an ACTH antagonist for therapeutic purposes. The conditions in which there is a potential clinical indication are relatively uncommon, and alternative therapies are well described in each case. However as a refinement to existing therapies or for the treatment of particularly difficult or complex cases, there would be a real clinical benefit. We have not considered a number of more common conditions, such as depressive illness or septic shock in which there might ultimately be a role for an ACTH antagonist, although these have been considered elsewhere (64).

The evidence suggests that progress is being made on more than one front in developing an antagonist. This has been delayed for many years by the absence of “clean” systems in which to test candidate peptides and compounds, owing to the problems in expressing the MC2R. This should no longer be a problem as a result of the identification of MRAP that enables cell surface expression of the MC2R. As a result there does seem to be a growing interest in this area and the next decade may witness exciting developments.

## Author Contributions

This work is part of the result of a 5-year project. Over this time, all authors have contributed information, ideas, and data to the work. The manuscript was primarily written by AC, with comment and editing from the other authors.

## Conflict of Interest Statement

The authors declare that the research was conducted in the absence of any commercial or financial relationships that could be construed as a potential conflict of interest.

## References

[1] Lorimer AR Dunn FG Jones JV Lawrie TD Beta-adrenoreceptor blockade in hypertension. Am J Med (1976) 60:877–85. 10.1016/0002-9343(76)90908-6 14502

[2] Siepler JK Campagna KD Donahue PE Bombeck CT. H2 receptor antagonists. Am J Hosp Pharm (1978) 35:141–5. 24338

[3] Osborne CK Tamoxifen in the treatment of breast cancer. N Engl J Med (1998) 339:1609–18. 10.1056/NEJM199811263392207 9828250

[4] Seferovic PM Pelliccia F Zivkovic I Ristic A Lalic N Seferovic J Mineralocorticoid receptor antagonists, a class beyond spironolactone – focus on the special pharmacologic properties of eplerenone. Int J Cardiol (2015) 200:3–7. 10.1016/j.ijcard.2015.02.096 26404746

[5] Singh SM Gauthier S Labrie F. Androgen receptor antagonists (antiandrogens): structure-activity relationships. Curr Med Chem (2000) 7:211–47. 10.2174/0929867003375371 10637363

[6] Capatina C Wass JA. 60 years of neuroendocrinology: acromegaly. J Endocrinol (2015) 226:T141–60. 10.1530/JOE-15-0109 26136383

[7] Peri A. Clinical review: the use of vaptans in clinical endocrinology. J Clin Endocrinol Metab (2013) 2013(98):1321–32. 10.1210/jc.2012-4082 23401044

[8] Clark AJ. 60 years of POMC: the proopiomelanocortin gene: discovery, deletion and disease. J Mol Endocrinol (2016) 56(4):T27–37. 10.1530/JME-15-0268 26643913

[9] Chretien M Mbikay M 60 years of POMC: from the prohormone theory to proopiomelanocortin and to proprotein convertases (PCSK1 to PCSK9). J Mol Endocrinol (2016) 56(4):T49–62. 10.1530/JME-15-0261 26762158

[10] Lowry P. 60 years of POMC: purification and biological characterisation of melanotrophins and corticotrophins. J Mol Endocrinol (2016) 56(4):T1–12. 10.1530/JME-15-0260 26643914

[11] Lim CT Grossman A Khoo B Normal physiology of ACTH and GH release in the hypothalamus and anterior pituitary in man. In: De Groot LJ Beck-Peccoz P Chrousos G Dungan K Grossman A Hershman JM, editors. Endotext [Internet]. South Dartmouth, MA: MDText.com, Inc. (2014).

[12] Schöneshöfer M Goverde HJ Corticotropin in human plasma. General considerations. Surv Immunol Res (1984) 3:55–63. 6326236 10.1007/BF02918598

[13] Ramachandrappa S Gorrigan RJ Clark AJ Chan LF. The melanocortin receptors and their accessory proteins. Front Endocrinol (Lausanne) (2013) 4:9. 10.3389/fendo.2013.00009 23404466 PMC3567503

[14] Fridmanis D Petrovska R Kalnina I Slaidina M Peculis R Schiöth HB Identification of domains responsible for specific membrane transport and ligand specificity of the ACTH receptor (MC2R). Mol Cell Endocrinol (2010) 321:175–83. 10.1016/j.mce.2010.02.032 20206229

[15] Veo K Reinick C Liang L Moser E Angleson JK Dores RM. Observations on the ligand selectivity of the melanocortin 2 receptor. Gen Comp Endocrinol (2011) 172:3–9. 10.1016/j.ygcen.2011.04.006 21501611

[16] Draper N Stewart PM. 11beta-hydroxysteroid dehydrogenase and the pre-receptor regulation of corticosteroid hormone action. J Endocrinol (2005) 186:251–71. 10.1677/joe.1.06019 16079253

[17] Keller-Wood M Hypothalamic-pituitary – adrenal axis-feedback control. Compr Physiol (2015) 5:1161–82. 10.1002/cphy.c140065 26140713

[18] Biller BM Grossman AB Stewart PM Melmed S Bertagna X Bertherat J Treatment of adrenocorticotropin-dependent Cushing’s syndrome: a consensus statement. J Clin Endocrinol Metab (2008) 93:2454–62. 10.1210/jc.2007-2734 18413427 PMC3214276

[19] Beardwell CG Adamson AR Shalet SM. Prolonged remission in florid Cushing’s syndrome following metyrapone treatment. Clin Endocrinol (Oxf) (1981) 14:485–92. 10.1111/j.1365-2265.1981.tb00638.x 6273022

[20] Boscaro M Sonino N Rampazzo A Mantero F. Response of pituitary-adrenal axis to corticotrophin releasing hormone in patients with Cushing’s disease before and after ketoconazole treatment. Clin Endocrinol (Oxf) (1987) 27:461–7. 10.1111/j.1365-2265.1987.tb01174.x 2830063

[21] Sonino N The use of ketoconazole as an inhibitor of steroid production. N Engl J Med (1987) 317:812–8. 10.1056/NEJM198709243171307 3306384

[22] Colao A Petersenn S Newell-Price J Findling JW Gu F Maldonado M A 12-month phase 3 study of pasireotide in Cushing’s disease. N Engl J Med (2012) 366:914–24. 10.1056/NEJMoa1105743 22397653

[23] Verhelst JA Trainer PJ Howlett TA Perry L Rees LH Grossman AB Short and long-term responses to metyrapone in the medical management of 91 patients with Cushing’s syndrome. Clin Endocrinol (Oxf) (1991) 35:169–78. 10.1111/j.1365-2265.1991.tb03517.x 1657460

[24] Juszczak A Ertorer ME Grossman A. The therapy of Cushing’s disease in adults and children: an update. Horm Metab Res (2013) 45:109–17. 10.1055/s-0032-1330009 23225246

[25] Miller WL Clinical review 54: genetics, diagnosis, and management of 21-hydroxylase deficiency. J Clin Endocrinol Metab (1994) 78:241–6. 10.1210/jc.78.2.241 8106606

[26] Nimkarn S Lin-Su K New MI. Steroid 21 hydroxylase deficiency congenital adrenal hyperplasia. Endocrinol Metab Clin North Am (2009) 38:699–718. 10.1016/j.ecl.2009.08.001 19944288

[27] Joint LWPES/ESPE CAH Working Group. Consensus statement on 21-hydroxylase deficiency from the Lawson Wilkins Pediatric Endocrine Society and the European Society for Paediatric Endocrinology. J Clin Endocrinol Metab (2002) 87:4048–53. 10.1210/jc.2002-020611 12213842

[28] Han TS Conway GS Willis DS Krone N Rees DA Stimson RH Relationship between final height and health outcomes in adults with congenital adrenal hyperplasia: United Kingdom congenital adrenal hyperplasia adult study executive (CaHASE). J Clin Endocrinol Metab (2014) 99:E1547–55. 10.1210/jc.2014-1486 24878054

[29] Ryan CJ Smith MR Fizazi K Saad F Mulders PF Sternberg CN Abiraterone acetate plus prednisone versus placebo plus prednisone in chemotherapy-naive men with metastatic castration-resistant prostate cancer (COU-AA-302): final overall survival analysis of a randomised, double-blind, placebo-controlled phase 3 study. Lancet Oncol (2015) 16:152–60. 10.1016/S1470-2045(14)71205-7 25601341

[30] Attard G Reid AH Auchus RJ Hughes BA Cassidy AM Thompson E Clinical and biochemical consequences of CYP17A1 inhibition with abiraterone given with and without exogenous glucocorticoids in castrate men with advanced prostate cancer. J Clin Endocrinol Metab (2012) 97:507–16. 10.1210/jc.2011-2189 22170708

[31] Kola B Grossman AB. Dynamic testing in Cushing’s syndrome. Pituitary (2008) 11:155–62. 10.1007/s11102-007-0079-x 18034306

[32] Mountjoy KG Robbins LS Mortrud MT Cone RD. The cloning of a family of genes that encode the melanocortin receptors. Science (1992) 257:1248–51. 10.1126/science.1325670 1325670

[33] Noon LA Franklin JM King PJ Goulding NJ Hunyady L Clark AJ. Failed export of the adrenocorticotrophin receptor from the endoplasmic reticulum in non-adrenal cells: evidence in support of a requirement for a specific adrenal accessory factor. J Endocrinol (2002) 174:17–25. 10.1677/joe.0.1740017 12098659

[34] Metherell LA Chapple JP Cooray S David A Becker C Rüschendorf F Mutations in MRAP, encoding a new interacting partner of the ACTH receptor, cause familial glucocorticoid deficiency type 2. Nat Genet (2005) 37:166–70. 10.1038/ng1501 15654338

[35] Webb TR Clark AJ. Minireview: the melanocortin 2 receptor accessory proteins. Mol Endocrinol (2010) 24:475–84. 10.1210/me.2009-0283 19855089 PMC5419097

[36] Sebag JA Hinkle PM. Melanocortin-2 receptor accessory protein MRAP forms antiparallel homodimers. Proc Natl Acad Sci U S A (2007) 104:20244–9. 10.1073/pnas.0708916105 18077336 PMC2154416

[37] Cooray SN Almiro Do Vale I Leung KY Webb TR Chapple JP The melanocortin 2 receptor accessory protein exists as a homodimer and is essential for the function of the melanocortin 2 receptor in the mouse y1 cell line. Endocrinology (2008) 149:1935–41. 10.1210/en.2007-1463 18162519

[38] Cooray SN Chung TT Mazhar K Szidonya L Clark AJ. Bioluminescence resonance energy transfer reveals the adrenocorticotropin (ACTH)-induced conformational change of the activated ACTH receptor complex in living cells. Endocrinology (2011) 152:495–502. 10.1210/en.2010-1053 21177829 PMC3058915

[39] Schwyzer R Chemistry and metabolic action of nonsteroid hormones. Ann Rev Biochem (1964) 33:259–86. 10.1146/annurev.bi.33.070164.001355 14268835

[40] Schwyzer R ACTH: a short introductory review. Ann N Y Acad Sci (1977) 297:3–26. 10.1111/j.1749-6632.1977.tb41843.x 211904

[41] Pogozheva ID Chai BX Lomize AL Fong TM Weinberg DH Nargund RP Interactions of human melanocortin 4 receptor with nonpeptide and peptide agonists. Biochemistry (2005) 44:11329–41. 10.1021/bi0501840 16114870 PMC2532597

[42] Yang Y Mishra VK Chen M Duffee E Dimmitt R Harmon CM. Molecular characterization of human melanocortin-5 receptor ligand-receptor interaction. Biochemistry (2013) 52:1737–45. 10.1021/bi3013593 23414113

[43] Hofmann K Chemistry and function of polypeptide hormones. Annu Rev Biochem (1962) 31:213–46. 10.1146/annurev.bi.31.070162.001241 13908175

[44] Eberle A Schwyzer R Hormone-receptor interactions. Demonstration of two message sequences (active sites) in alpha-melanotropin. Helv Chim Acta (1975) 58:1528–35. 10.1002/hlca.19750580604 170236

[45] Samuels ME Gallo-Payet N Pinard S Hasselmann C Magne F Patry L. Bioinactive ACTH causing glucocorticoid deficiency. Clin Endocrinol Metab (2013) 98:736–42. 10.1210/jc.2012-3199 23293326

[46] Schwyzer R. Structure and function in neuropeptides. Proc R Soc Lond B Biol Sci (1980) 210:5–20. 10.1098/rspb.1980.0115 6107931

[47] Chung TT Webb TR Chan LF Cooray SN Metherell LA King PJ The majority of adrenocorticotropin receptor (melanocortin 2 receptor) mutations found in familial glucocorticoid deficiency type 1 lead to defective trafficking of the receptor to the cell surface. J Clin Endocrinol Metab (2008) 93:4948–54. 10.1210/jc.2008-1744 18840636 PMC2635546

[48] Malik S Dolan TM Maben ZJ Hinkle PM. Adrenocorticotropic hormone (ACTH) responses require actions of the melanocortin-2 receptor accessory protein on the extracellular surface of the plasma membrane. J Biol Chem (2015) 290:27972–85. 10.1074/jbc.M115.668491 26424796 PMC4646036

[49] Seelig S Sayers G Schwyzer R Schiller P Isolated adrenal cells: ACTH(11-24), a competitive antagonist of ACTH(1-39) and ACTH(1-10). FEBS Lett (1971) 19:232–4. 10.1016/0014-5793(71)80521-5 11946219

[50] Seelig S Sayers G Isolated adrenal cortex cells: ACTH agonists, partial agonists, antagonists; cyclic AMP and corticosterone production. Arch Biochem Biophys (1973) 154:230–9. 10.1016/0003-9861(73)90053-2 4347679

[51] Li CH Chung D Yamashiro D Lee CY. Isolation, characterization, and synthesis of a corticotropin-inhibiting peptide from human pituitary glands. Proc Natl Acad Sci U S A (1978) 75:4306–9. 10.1073/pnas.75.9.4306 212744 PMC336102

[52] Szalay KS De Wied D Stark E. Effects of ACTH-(11-24) on the corticosteroid production of isolated adrenocortical cells. J Steroid Biochem (1989) 32:259–62. 10.1016/0022-4731(89)90261-6 2537913

[53] Goverde HJ Smals AG. The anomalous effect of some ACTH-fragments missing the amino acid sequence 1-10 on the corticosteroidogenesis in purified isolated rat adrenal cells. FEBS Lett (1984) 173:23–6. 10.1016/0014-5793(84)81009-1 6086397

[54] Kovalitskaia IA Kolobov AA Kampe-Nemm EA Iurovskií VV Sadovnikov VB Lipkin VM Synthetic peptide KKRR corresponding to the human ACTH fragment 15-18 is an antagonist of the ACTH receptor. Bioorg Khim (2008) 34:29–35. 18365734

[55] Hofmann K Montibeller JA Finn FM. ACTH antagonists. Proc Natl Acad Sci U S A (1974) 71:80–3. 10.1073/pnas.71.1.80 4359333 PMC387936

[56] Liang L Angleson JK Dores RM. Using the human melanocortin-2 receptor as a model for analyzing hormone/receptor interactions between a mammalian MC2 receptor and ACTH(1-24). Gen Comp Endocrinol (2013) 181:203–10. 10.1016/j.ygcen.2012.11.011 23201148

[57] Dores RM ACTH Antagonist Peptides. US Patent application US 2012/0309696 A1 (2012).

[58] Bouw E Huisman M Neggers SJ Themmen AP van der Lely AJ Delhanty PJ. Development of potent selective competitive-antagonists of the melanocortin type 2 receptor. Mol Cell Endocrinol (2014) 394:99–104. 10.1016/j.mce.2014.07.003 25017734

[59] Nensey NK Bodager J Gehrand AL Raff H. Effect of novel melanocortin type 2 receptor antagonists on the corticosterone response to ACTH in the neonatal rat adrenal gland in vivo and in vitro. Front Endocrinol (Lausanne). (2016) 7:23. 10.3389/fendo.2016.00023 27047449 PMC4800183

[60] Halem HA Ufret M Jewett I Mattei A Bastille A Beech J In vivo suppression of corticosterone in rodent models of Cushing’s disease with a selective, peptide MC2 receptor antagonist. Abstract presented to the Endocrine Society Annual Meeting, Boston (2016).

[61] Fani L Bak S Delhanty P van Rossum EF van den Akker EL. The melanocortin-4 receptor as target for obesity treatment: a systematic review of emerging pharmacological therapeutic options. Int J Obes (Lond) (2014) 38:163–9. 10.1038/ijo.2013.80 23774329

[62] Feldhaus AL Anderson K Dutzar B Ojala E McNeill PD Fan P A novel anti-ACTH antibody (ALD1613) neutralizes ACTH activity and reduced glucocorticoids in rats and nonhuman primates. Abstract presented to the Endocrine Society Annual Meeting, Boston (2016).

[63] Zhu QZ Hu J Mulay S Esch F Shimasaki S Solomon S. Isolation and structure of corticostatin peptides from rabbit fetal and adult lung. Proc Natl Acad Sci U S A (1988) 85:592–6. 10.1073/pnas.85.2.592 2829194 PMC279597

[64] Clark AJ Metherell LA. Mechanisms of disease: the adrenocorticotropin receptor and disease. Nat Clin Pract Endocrinol Metab (2006) 2:282–90. 10.1038/ncpendmet0165 16932299

